# Dendrite arborization requires the dynein cofactor NudE

**DOI:** 10.1242/jcs.170316

**Published:** 2015-06-01

**Authors:** Ashley L. Arthur, Sihui Z. Yang, Allison M. Abellaneda, Jill Wildonger

**Affiliations:** 1 Department of Biochemistry, University of Wisconsin-Madison, Madison, WI 53706, USA; 2 Graduate Program in Cellular and Molecular Biology, University of Wisconsin-Madison, Madison, WI 53706, USA; 3 Biochemistry Scholars Program, University of Wisconsin-Madison, Madison, WI 53706, USA

**Keywords:** Dendrite patterning, NudE, Nde1, Ndel1, Dynein, Microtubules, *Drosophila*

## Abstract

The microtubule-based molecular motor dynein is essential for proper neuronal morphogenesis. Dynein activity is regulated by cofactors, and the role(s) of these cofactors in shaping neuronal structure are still being elucidated. Using *Drosophila melanogaster*, we reveal that the loss of the dynein cofactor NudE results in abnormal dendrite arborization. Our data show that NudE associates with Golgi outposts, which mediate dendrite branching, suggesting that NudE normally influences dendrite patterning by regulating Golgi outpost transport. Neurons lacking NudE also have increased microtubule dynamics, reflecting a change in microtubule stability that is likely to also contribute to abnormal dendrite growth and branching. These defects in dendritogenesis are rescued by elevating levels of Lis1, another dynein cofactor that interacts with NudE as part of a tripartite complex. Our data further show that the NudE C-terminus is dispensable for dendrite morphogenesis and is likely to modulate NudE activity. We propose that a key function of NudE is to enhance an interaction between Lis1 and dynein that is crucial for motor activity and dendrite architecture.

## INTRODUCTION

Neuronal morphology is integral to neuronal function, affecting both the inputs that a neuron receives and the pattern of connectivity. Integral to neuronal morphology is the microtubule cytoskeleton, which provides structure to neurons and serves as a ‘track’ for intracellular transport that is mediated by molecular motors. Although recent studies have uncovered pivotal roles for the microtubule-based motors kinesin and dynein in neuronal morphogenesis, the mechanisms that regulate the behavior of molecular motors to generate precise and diverse neuronal morphologies are still being elucidated. In addition to mediating the transport of diverse cargo, dynein and kinesin have also been shown to regulate the orientation of microtubules in axons and dendrites (Kapitein and Hoogenraad, 2011; Kapitein et al., 2010; Lin et al., 2012; Yan et al., 2013; Zheng et al., 2008). Yet, it remains largely unknown how motor function is regulated to carry out different activities within a single neuron. To gain mechanistic insight into this question, we focused on dynein, the activity of which is regulated by different cofactors. One of these cofactors is the family of evolutionarily conserved nuclear distribution E (NudE) proteins. The vertebrate NudE family members Nde1 and Ndel1 (also known as NudEL) have been implicated in the regulation of early neurite extension (Bradshaw et al., 2013; Chansard et al., 2011; Mori et al., 2009; Yamada et al., 2010); however, it is currently unknown whether NudE family members have a role in dendrite morphogenesis.

To determine whether NudE has an integral role in neuronal morphogenesis, we turned to the fruit fly *Drosophila melanogaster* as a model for several reasons. First, mammals express both NudE and NudEL, which have been shown to act redundantly in multiple contexts, including neuronal development (Bradshaw et al., 2013), whereas flies have a single *nudE* gene, simplifying *in vivo* analysis. Second, loss of NudE or NudEL disrupts neuronal proliferation and migration early during neuronal development in mammals (Feng and Walsh, 2004; Hippenmeyer, 2014; Pawlisz and Feng, 2011; Pawlisz et al., 2008), confounding the *in vivo* analysis of dendrite and axon morphogenesis, which occurs later. The fly dendritic arborization (da) neurons that we employ as a model do not migrate, which enables us to clearly analyze whether NudE has a role in establishing the mature structure of a neuron. Using *Drosophila*, we determined that NudE is necessary for dendrite arborization. The dendrite patterning phenotypes we observed in *nudE*^−^ neurons are enhanced by reducing dynein activity, indicating that NudE acts with dynein to mediate dendrite arborization. Our data support a model in which NudE promotes dendrite growth and branching by facilitating an interaction between dynein and Lis1, another key dynein cofactor that frequently interacts with NudE and dynein as part of a tripartite complex. Our data are also consistent with the NudE C-terminus modulating NudE activity in order to promote proper dendrite growth and branching. Golgi outposts, which have been previously implicated in dendrite patterning (Horton and Ehlers, 2003; Horton et al., 2005; Ori-McKenney et al., 2012; Ye et al., 2007; Zhou et al., 2014), colocalize with NudE, suggesting that NudE regulates dendrite arborization by mediating their transport. Our results indicate that the microtubule cytoskeleton is also affected by disruption of NudE function. We find that microtubule dynamics are increased in *nudE*^−^ neurons. Moreover, although the orientation of the dendritic microtubules is similar to that of wild type, axonal microtubules are no longer organized in a uniform plus-end-distal array; instead, they display a mixed polarity in the absence of NudE. These changes in the microtubule cytoskeleton are also likely to contribute to the morphological defects displayed by *nudE*^−^ neurons. Combined, our data show that NudE acts with Lis1 and dynein to control dendrite arborization.

## RESULTS

### The dynein cofactor NudE is necessary for neuronal morphogenesis

**Fig. 1.**
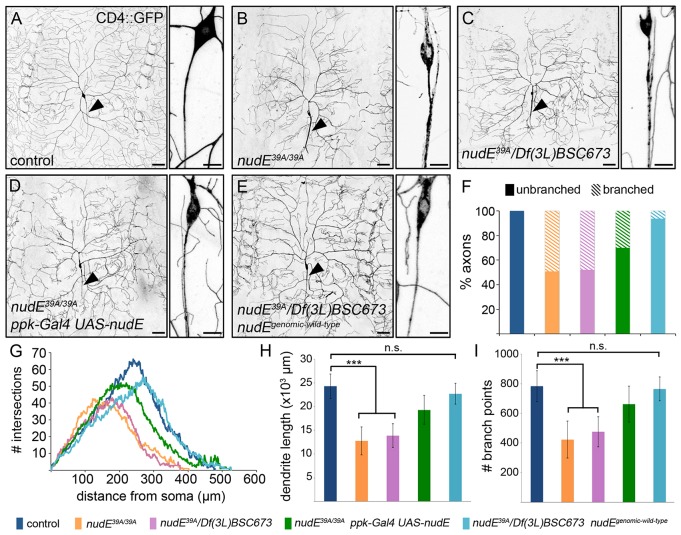
**Loss of NudE disrupts dendrite and axon morphogenesis.** (A–E) Representative images of class IV ddaC neurons illuminated by expression of *ppk-CD4–GFP* in live intact 3rd instar larvae. Arrowhead indicates axon. Scale bars: 50 µm (left-hand images); 10 µm (right-hand images showing magnified views of axon). (F–I) Quantification of axon branching (F), dendrite patterning (G), dendrite length (H) and dendrite branching (I) phenotypes. For *nudE^39A^*^/*39A*^ *ppk-Gal4 UAS-nudE* neurons, dendrite length was significantly different from that of control and *nudE^39A/39A^* neurons (*P*=0.003 and *P*=0.0004, respectively); the number of branch points was also significantly different from those of control and *nudE^39A/39A^* neurons (*P*=0.05 and *P*=0.002, respectively). Error bars indicate s.d., ***P=0.001–0.0001; n.s., not significant; *n*=8 neurons for all genotypes except for *nudE^39A/39A^ n=*9. The color code for the genotypes is shown at the bottom of the figure.

To determine whether the loss of NudE disrupts axon and/or dendrite morphogenesis, we utilized the class IV da neurons in *Drosophila* as a model. Located just beneath the transparent larval cuticle, the class IV da neurons are easily accessible for live-imaging analysis of neuronal morphology, as well as dynamic events such as microtubule growth and intracellular transport. Similar to the majority of mammalian neurons, the axons and dendrites of class IV neurons are morphologically distinct and emanate from opposite sides of the neuronal cell body. We first compared the morphology of the class IV ddaC neurons in wild-type 3rd instar larvae with ddaC neurons in larvae lacking NudE (*nudE^39A^*^/*39A*^ and *nudE^39A^*/*Df(3L)BSC673*; *nudE^39A^* is a protein null allele) (Wainman et al., 2009). Animals that lack NudE survive through late larval stages, making it possible to analyze ddaC neurons in homozygous mutant 3rd instar larvae. To visualize class IV da neuron morphology, we utilized green fluorescent protein (GFP)-tagged CD4 (CD4–GFP) expressed under the control of the *pickpocket* (*ppk*) enhancer (*ppk-CD4–GFP*) (Han et al., 2011). The loss of NudE resulted in a significant decrease in dendrite length and branch number in both the *nudE^39A^*^/*39A*^ and *nudE^39A^*/*Df(3L)BSC673* 3rd instar larvae (Fig. 1A–C,G–I). In addition, the dendrites within the distal arbors of *nudE*^−^ neurons had fewer branches in comparison with control ddaC neurons (Fig. 1G). Neurons lacking NudE also displayed a severe ‘axon splitting’ phenotype. Control axons emerged from the ddaC cell bodies as a single process that extended unbranched into the ventral nerve cord, whereas the axons projected by neurons lacking NudE formed multiple fine branches a short distance from the soma (Fig. 1B,C). Confocal optical sectioning revealed that these fine branches were indeed derived from the axons and were not just dendrite branches that bundled with the axons (data not shown). Some of these ectopic axon branches even grew back past the soma and occasionally invaded the dendritic arbor. Combined, these data reveal that NudE is necessary for normal dendrite growth and arbor patterning, as well as axonal morphogenesis.

To confirm that the axon and dendrite phenotypes are due to the loss of NudE, we generated two NudE transgenes – a genomic construct that encompassed the entire coding region of the *nudE* gene (*nudE^genomic-wild-type^*) and a UAS transgene (*UAS-nudE^wild-type^*). *nudE^genomic-wild-type^* rescued the lethality associated with loss of NudE, and the adults were morphologically normal, with the exception of slightly disordered anterior wing margin bristles. Dendrite length and branch number were fully rescued by *nudE^genomic-wild-type^* (Fig. 1E,G–I). *ppk-Gal4*, which is expressed in class IV da neurons starting in late stage embryos, was used to express *UAS-nudE^wild-type^.* In animals lacking NudE, *ppk-Gal4 UAS-nudE^wild-type^* only partially rescued the dendrite growth and branching defects (Fig. 1D,G–I). It is possible that *ppk-Gal4 UAS-nudE^wild-type^* failed to fully rescue dendrite arborization in *nudE*^−^ neurons owing to the timing and/or pattern of *ppk-Gal4*-mediated expression. However, expression of *UAS-nudE^wild-type^* utilizing other *Gal4* lines, including the ubiquitously expressed *actin-Gal4* and pan-neuronal *elav-Gal4*, which drive early expression, also failed to fully rescue the neuronal morphology defects and lethality (data not shown). Based on these results, it is likely that *UAS-nudE* might express NudE at suboptimal levels. It is also important to note that overexpressing *nudE* in control animals, using either *UAS-nudE* or *nudE^genomic-wild-type^*, had no effect on viability or neuronal morphology, which indicates that increasing expression of NudE is not disruptive.

**Fig. 2.**
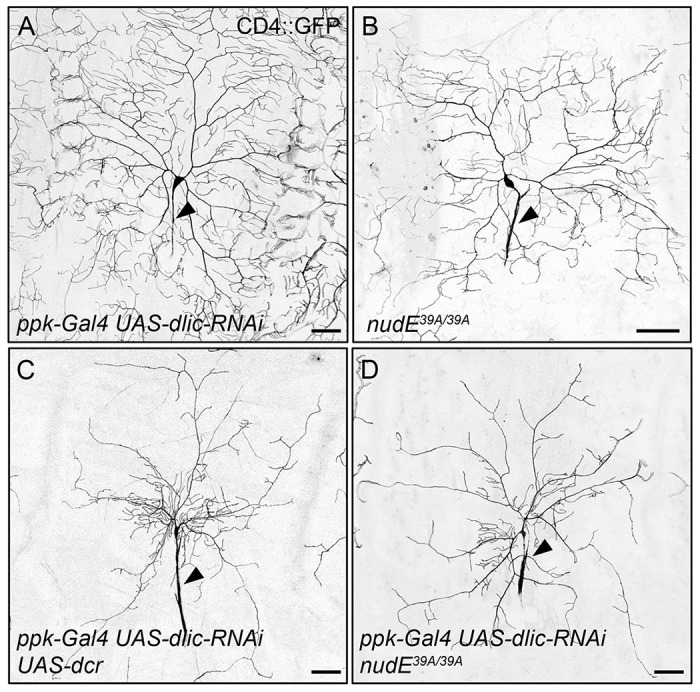
**Loss of NudE enhances the dynein loss-of-function dendrite morphogenesis phenotype.** (A–D) Representative images of class IV ddaC neurons illuminated by expression of *ppk-CD4–GFP* in live intact 3rd instar larvae. Dendrite patterning was mildly disrupted in *ppk-Gal4 UAS-dlic-RNAi* larvae (A). The expression of *dcr* enhances the dendrite arborization phenotype caused by reducing levels of Dlic (C). Reducing levels of NudE and Dlic at the same time reduces dendrite growth and branching, similar to *ppk-Gal4 UAS-dlic-RNAi UAS-dcr* (D). Arrowheads indicate axons. Scale bars: 50 µm.

We next set out to determine whether the change in dendrite arborization that resulted from the loss of NudE was due to decreased dynein function. In order to do so, we assayed whether *nudE* genetically interacts with *dynein light intermediate chain* (*dlic*), which encodes an essential subunit of the dynein complex. RNA interference (RNAi)-mediated knockdown of *dlic* produced a very mild dynein loss-of-function dendrite phenotype, which could be enhanced by the co-expression of *dicer2* (*dcr*) to generate a dendritic arbor that resembled those found in animals with loss of dynein function (Fig. 2A,C) (Satoh et al., 2008; Zheng et al., 2008). The *dlic-RNAi* dendrite-patterning phenotype was enhanced by the loss of NudE (Fig. 2D), indicating that NudE acts with dynein in order to mediate dendrite growth and branching.

### NudE associates with Golgi outposts in dendrites

NudE family members have been previously implicated in axonal transport in mammalian neurons, as well as in fruit fly motor neurons, raising the possibility that the dendrite phenotypes that we observed in *nudE*^−^ neurons are caused by perturbation of the transport of vesicles and/or organelles in dendrites (Liang et al., 2004; Pandey and Smith, 2011; Segal et al., 2012; Shao et al., 2013; Shim et al., 2008; Shmueli et al., 2010; Valakh et al., 2012; Yamada et al., 2008; Yi et al., 2011; Zhang et al., 2009). In neurons, a subset of Golgi complexes localizes to dendrites in the form of ‘outposts’, and these Golgi outposts have been shown to mediate dendrite arborization and microtubule growth (Horton and Ehlers, 2003; Horton et al., 2005; Ori-McKenney et al., 2012; Ye et al., 2007). Although previous studies have demonstrated that the loss of dynein activity changes the distribution Golgi outposts in ddaC neurons (Zheng et al., 2008), it is still unknown which dynein cofactors participate in the transport of Golgi outposts, and how cofactors affect Golgi outpost dynamics, localization and/or function.

**Fig. 3.**
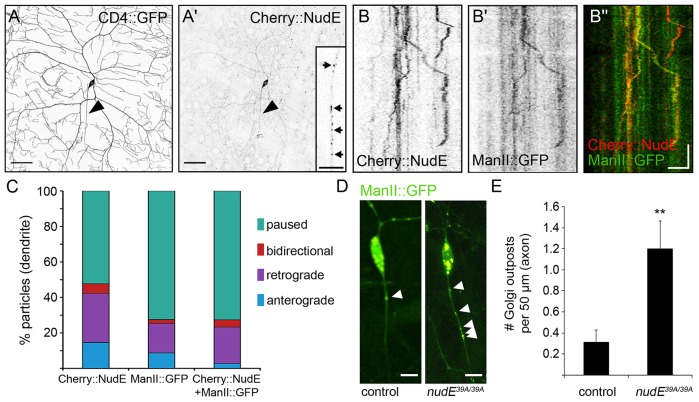
**NudE colocalizes with Golgi outposts in dendrites and prevents them from entering axons.** (A,A′) Representative image of a ddaC neuron co-expressing CD4–GFP (A) and Cherry–NudE (A′). Arrowhead indicates axon. Inset in A′ shows a magnified view of an axon, arrows indicate several Cherry–NudE particles. (B) Representative kymograph showing the motility of Cherry–NudE (B, red channel in B″) and the Golgi marker ManII–GFP (B′, green channel in B″) in dendrites. Cell body is to the left. (C) Quantification of Cherry–NudE and ManII–GFP movement and colocalization in dendrites (the distances that Cherry–NudE and ManII–GFP particles travel in the anterograde or retrograde direction are similar, data not shown). (D,E) Representative images (D) and quantification (E) of Golgi outposts in the axons of control (*ppk-Gal4 UAS-ManII–GFP*, left panel in D) and *nudE^39A/39A^* (right panel in D) neurons. Arrowheads indicate Golgi outposts. Error bars indicate s.d., **P=0.01–0.001, *n*=19 control axons, *n*=15 *nudE^39A/39A^* axons. Scale bars: 50 µm (A); 10 µm (D, inset of A′); 5 µm (*x* axis of B″); 30 s (*y* axis of B″).

To test the idea that NudE mediates Golgi outpost localization and/or function, we set out to determine whether NudE usually associates with Golgi outposts in dendrites. To do so, we first generated a construct to express a fluorescently-tagged NudE protein (*UAS-Cherry–NudE*) in neurons and monitored its dynamic localization by using live-cell imaging. When expressed in class IV da neurons, Cherry–NudE localized to both dendrites and axons, and had a punctate appearance typical of vesicle-associated proteins (Fig. 3A,A′). Cherry–NudE particles moved in anterograde and retrograde directions at speeds typical of kinesin- and dynein-mediated transport (0.66±0.39 µm s^−1^ in axons and 0.48±0.22 µm s^−1^ in dendrites). Consistent with a role in axonal transport, we observed that Cherry–NudE colocalized with the synaptic vesicle marker synaptobrevin–GFP, as well as with mitochondria-targeted GFP (mito–GFP; data not shown), indicating that NudE participates in the transport of multiple different types of cargo in neurons. Next, we utilized the medial-Golgi marker ManII–GFP, which labels both Golgi and Golgi outposts, in combination with Cherry–NudE (*Gal4^477^ UAS-ManII–GFP UAS-Cherry–NudE*; *Gal4^477^* is expressed in class IV da neurons). Live-imaging analysis revealed that Cherry–NudE and ManII–GFP colocalized in dendrites (Fig. 3B–Bʹʹ). To investigate whether NudE participates in a specific aspect of Golgi outpost localization, we characterized Cherry–NudE and ManII–GFP colocalization. Nearly two-thirds of the Cherry–NudE and ManII–GFP particles that colocalized were stationary (Fig. 3C), suggesting that NudE primes Golgi outposts for transport and/or that they associate with Golgi outposts at the end of runs. We found that the majority of dynamic colocalized Cherry–NudE and ManII–GFP particles traveled in a retrograde direction, indicative of movement towards the microtubule plus end and kinesin-mediated transport (Fig. 3C). Fewer than 5% of the colocalized particles moved in a direction (anterograde) that was consistent with dynein-mediated transport (Fig. 3C). Indeed, only a few anterograde-moving Golgi outposts colocalized with Cherry–NudE, whereas the majority of Golgi outposts that moved in a retrograde direction colocalized with Cherry–NudE. This supports a model in which NudE acts to limit dynein activity during events such as plus-end-directed transport (e.g. when dynein is carried by kinesin to the axon terminal) and/or when dynein is stationary at the beginning and end of runs.

We also characterized how the loss of *nudE* disrupted the localization of Golgi outposts. More specifically, we sought to determine whether Golgi outposts mislocalize to axons in neurons that lack NudE, as previously reported for neurons with reduced dynein activity (Zheng et al., 2008). In *nudE^39A/39A^* neurons, the number of Golgi outposts that were present in axons increased significantly (Fig. 3D,E), consistent with the model that NudE is required for the dendrite-enriched localization of Golgi outposts.

### NudE modulates microtubule dynamics and is necessary for the uniform plus-end-distal polarity of axonal microtubules

Dendrite morphology is highly dependent on the microtubule cytoskeleton, raising the possibility that the dendrite arborization phenotypes resulting from the loss of NudE are also be associated with an underlying defect in the microtubule cytoskeleton. Microtubules have a plus- and minus-end, and growth occurs predominantly at the microtubule plus end. In the axons of da neurons, all microtubules are oriented with their plus ends positioned distal to the cell body, similar to mammalian neurons, whereas in dendrites, the majority of microtubule plus ends are located proximally (Rolls et al., 2007). To monitor microtubule growth dynamics and to determine the orientation of microtubules in axons and dendrites, we utilized EB1–GFP, which is a fusion of the microtubule-plus-end-binding protein EB1 with GFP (Rolls et al., 2007). EB1 associates with growing microtubule plus ends and dissociates a short distance from the plus-end tip; this behavior gives EB1–GFP a comet-like appearance in live-cell imaging experiments and enables us to simultaneously analyze both microtubule growth and microtubule orientation.

**Fig. 4.**
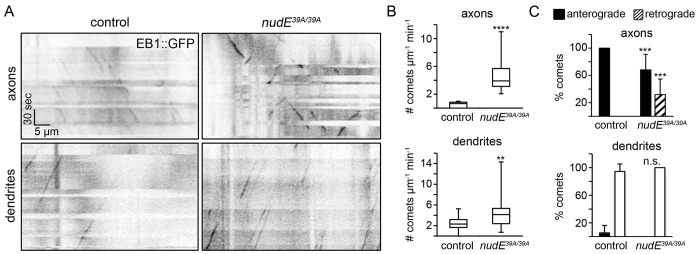
**Loss of NudE affects microtubule growth and the polarity of axonal but not dendritic microtubules.** (A) Kymographs generated from representative movies of EB1–GFP comets present in the axons (top) and dendrites (bottom) of neurons in live intact 3rd instar larvae. *ppk-EB1–GFP* control (left) and *nudE^39A/39A^* (right) neurons are shown. The cell body is to the left in all kymographs. Scale bars: 5 µm (*x* axis); 30 s (*y* axis). (B) Quantification of EB1–GFP comet frequency in axons (top) and dendrites (bottom). Comet frequency reflects the number of growing microtubules (control axons, *n*=13 neurons, which includes a total of 58 comets; *nudE^39A/39A^* axons, *n*=11 neurons, which includes a total of 156 comets; control dendrites, *n*=24 dendrite segments in nine neurons, which includes a total of 247 comets; *nudE^39A/39A^* dendrites, *n*=32 dendrite segments in eight neurons, which includes a total of 540 comets). Boxes represent first and third quartiles (median indicated by line) and whiskers indicate minimum and maximum values. (C) Quantification of the direction that EB1–GFP comets traveled, which reflects the polarity of microtubules in axons (top) and dendrites (bottom) (control axons, *n*=11 axons with 135 comets; control dendrites, *n*=12 dendrites with 323 comets; *nudE^39A/39A^* axons, *n*=9 axons with 115 comets; *nudE^39A/39A^* dendrites, *n*=8 dendrites with 540 comets). Error bars indicate s.d., **P=0.01–0.001, ***P=0.001–0.0001, ****P<0.0001; n.s., not significant.

In neurons lacking NudE, the frequency of EB1–GFP comets increased significantly in both axons and dendrites (Fig. 4A,B), indicating that NudE has a crucial role in regulating microtubule growth and dynamics. These data suggest that the dendrite growth and branching defects in *nudE*^−^ neurons might be due, in part, to an imbalance in microtubule growth and stability. Next, we investigated whether the loss of NudE affects the orientation of microtubules, similar to the loss of dynein function (Zheng et al., 2008). Our experiments using EB1–GFP revealed that the orientation of dendritic microtubules was not affected by the loss of NudE. In the dendrites of control neurons, as well as neurons lacking NudE, EB1–GFP comets traveled predominantly towards the cell body (Fig. 4A,C). However, the loss of NudE did affect the orientation of axonal microtubules. In the axons of control ddaC neurons, all EB1–GFP comets traveled away from the cell body, indicating that the microtubules were oriented with plus ends distal (Fig. 4A,C). By contrast, in the axons of ddaC neurons lacking NudE, EB1–GFP comets traveled both away from and towards the cell body, indicating that microtubule polarity was mixed (Fig. 4A,C). This is consistent with previous studies that have reported that the loss of dynein activity disrupts the organization of axonal microtubules (Baas and Mozgova, 2012; Zheng et al., 2008). To assess microtubule polarity, we also utilized Nod–GFP, a chimeric protein that translocates to the minus ends of microtubules (Andersen et al., 2005; Clark et al., 1997). When expressed in control and *nudE*^−^ ddaC neurons, Nod–GFP localized predominantly to dendrites, consistent with the minus-end distal organization of dendritic microtubules (data not shown). In contrast to controls, Nod–GFP mislocalized to the axons of a significant number of *nudE^39A/39A^* neurons. These results suggest that NudE specifically regulates the polarity of axonal, but not dendritic, microtubules and that NudE has a role in modulating microtubule dynamics in both compartments.

### Reducing γ-tubulin activity does not rescue the axonal microtubule polarity defect in *nudE*^−^ neurons

Our data revealed that neurons lacking *nudE* contain microtubules of mixed polarity, as well as ectopic Golgi outposts. The mislocalized Golgi outposts might serve as sites of ectopic microtubule nucleation in axons, resulting in microtubules that have their plus ends positioned proximally. Microtubules are nucleated at Golgi outposts and somatic Golgi by the microtubule nucleator γ-tubulin (Chabin-Brion et al., 2001; Efimov et al., 2007; Ori-McKenney et al., 2012; Rivero et al., 2009). We reasoned that if the Golgi outposts present in the axons of *nudE*^−^ neurons serve as ectopic sites for microtubule nucleation, knocking down γ-tubulin should restore the microtubules to a uniform plus-end-distal array. Knocking down γ-tubulin can also be used to test whether changes in γ-tubulin localization and/or activity in axons are generally responsible for the appearance of plus-end-proximal microtubules, irrespective of whether microtubules are nucleated at the ectopic axonal Golgi outposts. For example, it is also possible that NudE normally functions to exclude γ-tubulin from axons. Thus, these experiments were designed to resolve whether γ-tubulin-mediated nucleation is responsible for the change in axonal microtubule polarity that occurs in the absence of NudE.

**Fig. 5.**
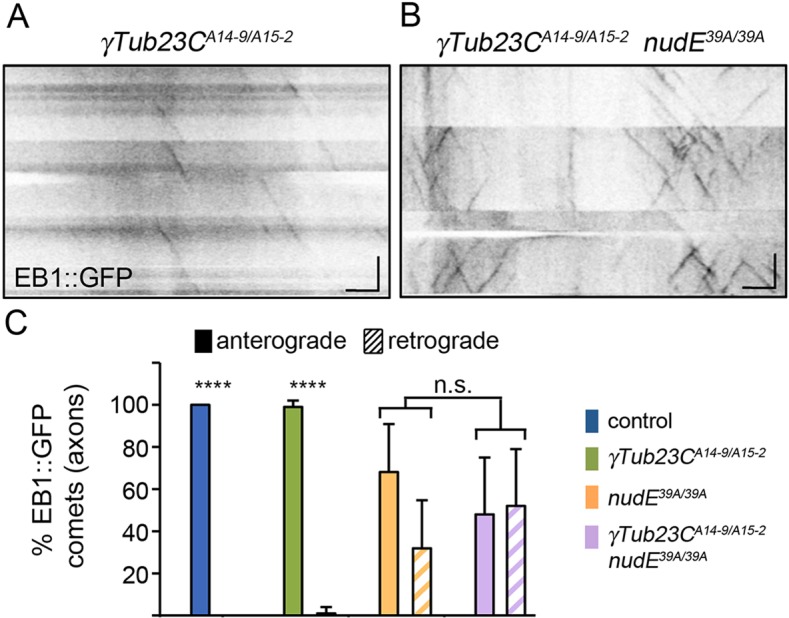
**γ-tubulin-mediated microtubule nucleation is not responsible for the change in axonal microtubule polarity** **that is** **caused by the loss of NudE.** (A,B) The polarity of axonal microtubules was determined using EB1–GFP, the comet trajectories of which are plotted in kymographs. The cell body is to the left in all kymographs. Scale bars: 5 µm (*x* axis); 30 s (*y* axis). (C) Quantification of the direction that EB1–GFP comets traveled in axons. *γTub23C^A14-9/A15-2^*, *n*=16 axons with 156 comets. *γTub23C^A15-2/A14-9^*; *nudE^39A/39A^*, *n*=12 axons with 193 comets. The percentage of comets traveling anterograde and retrograde in the axons of *nudE^39A^^/39A^* and *γTub23C^A15-2/A14-9^*; *nudE^39A/39A^* neurons was significantly different from control (****P<0.0001 for both genotypes). n.s., not significant.

To determine whether γ-tubulin-mediated nucleation contributes to the axonal microtubule-polarity defects in *nudE*^−^ neurons, we used the hypomorphic alleles γ*Tub23C^A15-2^* and γ*Tub23C^A14-9^* in combination, which allows the animals to survive to larval stages (we refer to this genetic combination hereafter as ‘γ-tubulin knockdown’). In the axons of γ-tubulin knockdown neurons, virtually all the EB1–GFP comets traveled in an anterograde direction, similar to that in control neurons (Fig. 5A,C). This indicates that decreasing the levels of γ-tubulin does not affect axonal microtubule polarity. Next, we knocked down γ-tubulin in animals that lacked NudE. Reducing γ-tubulin in *nudE*^−^ neurons did not change the number of retrograde EB1–GFP axonal comets in axons compared with that in *nudE*^−^ neurons (Fig. 5B,C). Thus, decreased γ-tubulin activity does not affect the number of retrograde EB1–GFP comets in the axons of neurons that lack NudE.

### The NudE C-terminus is not required for dendrite branching

Next, we sought to determine whether we could identify which domains of NudE are crucial for its activity in dendrite growth and branching. Members of the NudE family of proteins comprise an N-terminal coiled-coil domain and an unstructured, poorly conserved C-terminus. Sites in both the N- and C-terminus of NudE and NudEL have been shown to bind to different subunits of the dynein complex, and conserved residues in the coiled-coil domain have been shown to recruit another important dynein cofactor, Lis1 (Liang et al., 2004; Sasaki et al., 2000; Stehman et al., 2007; Wang and Zheng, 2011; Zyłkiewicz et al., 2011). The NudE and NudEL C-terminal domains also mediate several protein interactions and are targets of post-translational modifications, including phosphorylation (Bradshaw et al., 2013). The unphosphorylated C-terminal domain has been proposed to inhibit NudE and NudEL activity by folding back to interact with the N-terminus (Soares et al., 2012; Zyłkiewicz et al., 2011). Yet, how the C-terminal domain affects the activity of NudE-family members in cells is still poorly understood – some reports suggest that the C-terminal domain is essential (Mori et al., 2007; Stehman et al., 2007), whereas other reports indicate that it is dispensable (Wang and Zheng, 2011; Zyłkiewicz et al., 2011).

**Fig. 6.**
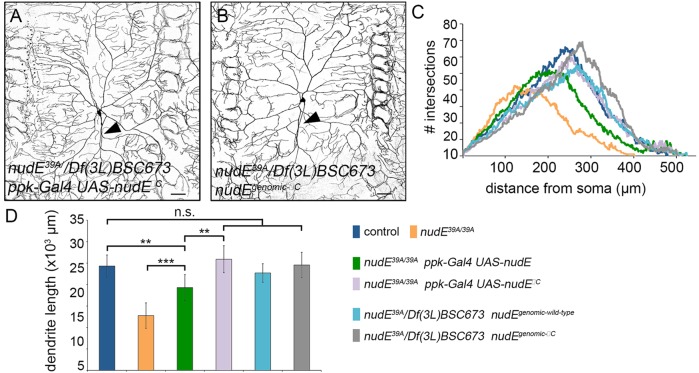
**The NudE N-terminus is sufficient for normal dendrite arborization.** (A,B) Representative images of class IV ddaC neurons illuminated through expression of *ppk-CD4–GFP* in live intact 3rd instar larvae. Arrowhead indicates axons. Scale bars: 50 µm. (C,D) Dendrite arborization was quantified using Sholl analysis (C) and by measuring dendrite length (D). Error bars indicate s.d., **P=0.01–0.001, ***P=0.001–0.0001; n.s., not significant; *n*=8 neurons for all genotypes, except *n=*9 for *nudE^39A/39A^*.

To determine whether the C-terminal domain of *Drosophila* NudE regulates and/or is necessary for its activity in neurons, we generated a construct in order to express a truncated NudE protein that lacked the entire C-terminal domain (*UAS-nudE^ΔC^*). As described above, expressing a wild-type NudE protein under the control of *ppk-Gal4* (*ppk-Gal4 UAS-nudE^wild type^*) only partially rescued the NudE loss-of-function phenotypes (Fig. 1D,G–I). This result suggested that we could utilize the Gal4-UAS system to identify both loss- and gain-of-function mutations in NudE. We reasoned that loss-of-function mutations in NudE would result in a weaker rescue phenotype than the wild-type protein, whereas gain-of-function mutations should fully rescue *nudE*^−^ neurons. The expression of NudE^ΔC^ in class IV da neurons (*ppk-Gal4 UAS-nudE^ΔC^*) fully rescued dendrite arborization defects in *nudE^39A/39A^* larvae, suggesting that removing the C-terminal domain increased this aspect of NudE activity (Fig. 6A,C,D). To further probe the role of the NudE C-terminal domain in neuronal morphogenesis and microtubule organization, we also generated a genomic *nudE* construct that lacked the C-terminal domain (*nudE^genomic-ΔC^*). Consistent with the experiments using the *UAS-nudE^ΔC^* transgene, *nudE^genomic-ΔC^* fully rescued the dendrite arborization defects in neurons that lacked NudE (Fig. 6B–D). These data are consistent with the model that the C-terminal domain of NudE has an inhibitory effect on the activity of the full-length NudE protein. However, these results also surprisingly reveal that the C-terminal domain, which is entirely removed in these experiments, is dispensable for dendrite growth and branching. Thus, these data indicate that during dendrite morphogenesis, the NudE C-terminal domain might act as an ‘activity switch’ but is otherwise redundant or unnecessary for dendrite growth and branching.

### Overexpression of Lis1 rescues dendrite arborization defects in *nudE*^−^ neurons

**Fig. 7.**
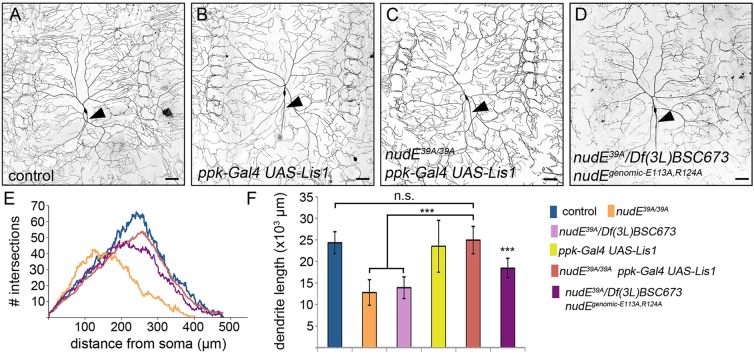
**Overexpressing Lis1 rescues abnormal dendrite arborization that is caused by the loss of NudE.** (A–D) Representative images of ddaC neurons illuminated through expression of *ppk-CD4–GFP* in live intact 3rd instar larvae. Arrowheads indicate axons. Scale bars: 50 µm. (E,F) Dendrite arborization was quantified using Sholl analysis (E) and by measuring dendrite length (F). Error bars indicate s.d., ***P=0.001–0.0001, n.s., not significant; *n*=8 neurons for all genotypes, except *n*=9 for *nudE^39A/39A^*.

One key molecular function that has been proposed for the NudE family of proteins is the promotion of an interaction between dynein and Lis1 (McKenney et al., 2010; Shu et al., 2004). Although Lis1 can directly interact with dynein, these reports show that NudE tethers Lis1 to dynein, prolonging the interaction between cofactor and motor (McKenney et al., 2010). Recent biochemical studies using purified proteins have shown that Lis1 directly regulates several properties of the dynein motor – such as the amount of force that the motor produces – that are likely to be crucial for its function in cells (Huang et al., 2012; McKenney et al., 2010). However, it is not known whether a principal function of NudE in neurons is to mediate and/or enhance an association between Lis1 and dynein during neuronal morphogenesis. Several groups have shown that increasing Lis1 concentrations can compensate, at least partially, for the loss of NudE or NudEL in different cell types and in cell extracts (Efimov, 2003; Li et al., 2005; Shu et al., 2004; Wang and Zheng, 2011; Zyłkiewicz et al., 2011). Based on these findings, we sought to determine whether increasing Lis1 levels in neurons rescues the dendrite arborization defects that manifest in *nudE*^−^ class IV da neurons. To overexpress Lis1, we utilized *ppk-Gal4* in combination with *UAS-Lis1* and then quantified the effects of Lis1 overexpression in both control and *nudE*^−^ neurons. First, increasing Lis1 levels in control ddaC neurons did not affect dendrite length, dendrite branch number or dendrite branch position, indicating that elevated Lis1 levels does not interfere with normal dendritogenesis (Fig. 7B,F). As described above, loss of NudE significantly reduced dendrite arbor size and resulted in an increased number of proximal branches. Increasing the amount of Lis1 in neurons that lacked NudE rescued these dendrite morphogenesis defects, resulting in neurons with normal dendrite length, branch number and branch distribution (Fig. 7C,E,F). These results raise the possibility that the dendrite arborization defects caused by the loss of NudE might be largely due to a decrease in Lis1 activity, which in turn results in less active dynein. As previously reported (Zheng et al., 2008), we also found that loss of Lis1 resulted in a severe dendrite morphogenesis defect that resembled the loss of dynein function (data not shown). To further probe the relationship between NudE and Lis1 in dendritogenesis, we generated a mutant genomic *nudE* construct that was designed to disrupt the interaction between these two proteins (*nudE^genomic-E113A,R124A^*). We mutated two amino acids (E113A and E124A) in the N-terminal domain of NudE that have been previously shown to diminish the interaction between NudE and Lis1 (Derewenda et al., 2007; Wang and Zheng, 2011; Zyłkiewicz et al., 2011). In contrast to *nudE^genomic-wild-type^*, *nudE^genomic-E113A,R130A^* only partially rescued the dendrite arborization defects in *nudE^39A^*^/*39A*^ animals, indicating that an interaction with Lis1 is necessary for NudE to mediate normal dendrite growth and branching (Fig. 3D–F). In summary, these results are consistent with a model in which NudE interacts with Lis1 and dynein to promote dendrite morphogenesis.

## DISCUSSION

Neuronal morphology is shaped by the activity of molecular motors, the function of which is regulated by cofactors. Yet how these cofactors influence neuronal architecture is still poorly understood. Although the importance of NudE-family members in the proliferation and migration of neuronal precursors has been established, it remains largely unknown whether and how NudE influences the morphogenesis of mature neurons (Bradshaw et al., 2013). In the developing vertebrate nervous system, determining whether NudE and/or the closely related NudEL act in dendrite morphogenesis is made difficult by functional redundancy between these two proteins. Also, dendrite extension occurs after neurons have migrated away from their birthplace, and neuronal migration is disrupted by the loss of either protein (Bradshaw et al., 2013). Although one study has reported abnormal dendrite arborization in mouse Purkinje cells that lacked NudEL, this defect could not be dissociated from impaired migration (Hippenmeyer et al., 2010). In *Drosophila*, there is only one *nudE* gene, and we utilized a class of neurons, the da neurons, that do not migrate away from their birthplace. Using the da neurons as a model system, our experiments reveal that NudE is necessary for normal dendrite patterning.

Based on the known functions of NudE-family members, we predicted that a central activity of NudE in neurons is to enhance the interaction between dynein and Lis1. NudE has been proposed to serve as a molecular tether between these proteins, thus enabling Lis1 to be effective even at low concentrations (McKenney et al., 2010). In support of this idea, other groups have demonstrated that the loss of NudE can be compensated for by elevating the amount of Lis1, which presumably increases the probability of interaction between Lis1 and dynein (Efimov, 2003; Li et al., 2005; Shu et al., 2004; Wang and Zheng, 2011; Zyłkiewicz et al., 2011). Our results show that increasing the level Lis1 in fly da neurons rescues the dendrite growth and branching defects that result from the loss of NudE, leading us to propose that NudE acts to facilitate an interaction between Lis1 and dynein that is crucial for dendrite morphogenesis. Although residues in both the N- and C-terminus of NudE have been shown to mediate interactions with Lis1 and dynein (Liang et al., 2004; Sasaki et al., 2000; Stehman et al., 2007), a recent study has revealed that a C-terminal-truncated NudE protein is able to interact with Lis1 and dynein simultaneously (Wang et al., 2013). Thus, the NudE C-terminus is not necessary in order for NudE to facilitate an interaction between Lis1 and dynein. Rather, the C-terminus might even impede the ability of NudE to act as a tether because the NudE C-terminal domain can fold-back to mask the N-terminal residues that mediate binding to Lis1 and dynein (Soares et al., 2012). One prediction based on these data is that removing the C-terminus serves to increase NudE activity, which is consistent with the results our experiments using the Gal4-UAS system, which showed that NudEΔC but not full-length NudE rescues dendrite growth defects. Although others have shown that NudE-mediated transport in axons can be regulated by the phosphorylation of residues in the NudE C-terminus (Pandey and Smith, 2011), our results demonstrate that dendrite growth and branching do not require the NudE C-terminus. This suggests that either phosphorylation of these C-terminal residues does not play a role in transport in dendrites or that phosphorylation is only necessary to make the N-terminal binding sites for Lis1 and/or dynein accessible, which can also be accomplished by deleting the C-terminus. Combined, our data are consistent with the model that a key function of NudE in dendrite arborization is to facilitate an interaction between Lis1 and dynein, and that the ability of NudE to perform this function can be modulated by its C-terminal domain.

A likely model is that NudE mediates the transport of cargo that are crucial for proper dendrite growth and branching, such as Golgi outposts. To explore this model, we utilized live-imaging to track the dynamic localization of fluorescently-tagged NudE (Cherry–NudE). Our results implicate NudE in the transport of different vesicles and organelles, consistent with previous findings (Bradshaw et al., 2013). Our live-imaging experiments also showed that Cherry–NudE colocalizes with ManII–GFP, providing evidence that NudE associates with Golgi outposts. The majority of Golgi outposts that were associated with NudE were stationary. This suggests that NudE might be required at the beginning and/or end of a transport event, when it might serve to regulate the interaction between Lis1 and dynein. Unfortunately, we were not able to visualize the expression of a *Lis1–GFP* transgene (unpublished data), which would have provided insight into whether NudE and Lis1 colocalize with moving and/or stationary cargo in dendrites. It is also possible that NudE is part of a complex that tethers stationary Golgi outposts to microtubules, but as NudE lacks a microtubule-binding domain, another protein would necessarily be involved in this case. Because stationary Golgi outposts have been shown to be sites of microtubule nucleation in dendrites (Ori-McKenney et al., 2012), it is interesting to speculate whether NudE plays a role in regulating microtubule growth by influencing Golgi outpost motility, although implicit in such a model is that outpost motility and microtubule nucleation are coordinated. Recent experiments by others have revealed that microtubule nucleation is also influenced by Golgi outpost organization – outposts with both medial- and trans-Golgi compartments are more likely to support microtubule nucleation than those comprising a single compartment (Zhou et al., 2014). Thus, it is possible that a change in Golgi outpost dynamics and/or organization might underlie the increase in microtubule dynamics that we observe in neurons lacking NudE. For example, previous studies have reported that loss of mammalian NudE proteins affect somatic Golgi organization and localization (Lam et al., 2010; Liang et al., 2004; Sasaki et al., 2005). However, additional information about the relationships between Golgi outpost motility, compartmentalization and microtubule nucleation must be obtained before this question can be resolved.

Our experiments also revealed that microtubule dynamics increased in both dendrites and axons in the absence of NudE. As regards to dendrite growth and branching, a likely model is that microtubule dynamics and stability must be balanced to produce a properly patterned arbor. The increase in EB1–GFP comets in *nudE*^−^ neurons is likely to reflect an increase in microtubule dynamics that does not allow normal dendrite growth and branching. A previous report has shown that loss of mammalian NudEL results in decreased microtubule dynamics at the axon hillock during neurite outgrowth (Mori et al., 2009). This finding in combination with our results suggests that NudE-family members might play different roles in microtubule growth and stability at different stages of neuronal morphogenesis. Previous studies have shown that dynein acts through a mechanical tension-based mechanism to directly regulate microtubule dynamics at the cell cortex, where microtubule plus ends face the cell membrane ‘end on’ (Grallert et al., 2006; Hendricks et al., 2012; Laan et al., 2012). More recently, it has been reported that p150^Glued^, which is a component of the dynein-interacting dynactin complex, also regulates microtubule stability, albeit through a different dynein-independent mechanism that involves an interaction with tubulin dimers and microtubules (Lazarus et al., 2013). However, the majority of dendritic and axonal microtubules are unlikely to be attached ‘end on’ to the neuronal cell cortex, and NudE does not have a (known) microtubule- or tubulin-binding domain. There is evidence that Lis1 can function as an anti-catastrophe factor *in vitro* through an unknown mechanism (Sapir et al., 1997). This suggests that NudE might be an essential component, or regulator, of a Lis1-containing complex that modulates microtubule dynamics.

Lastly, our data demonstrate that NudE, like dynein, is required for the uniform plus-end-distal array of axonal microtubules. NudE might regulate axonal microtubule polarity by affecting either the local nucleation and growth of microtubules in axons or the translocation of microtubules into axons. One possibility is that in the absence of NudE, microtubules are ectopically nucleated in axons, which creates a population of microtubules with their plus ends oriented towards the cell body. Our experiments reveal that reducing the activity of the microtubule nucleator γ-tubulin in *nudE*^−^ neurons fails to restore axonal microtubules to a uniform plus-end-distal orientation. A recent report has suggested that a decrease as well as an increase in γ-tubulin activity disrupts axonal microtubule polarity by stimulating local microtubule nucleation at atypical sites (Nguyen et al., 2014). Our data show that NudE colocalizes with Golgi outposts, which have been shown to be sites of γ-tubulin-dependent microtubule nucleation in dendrites (Ori-McKenney et al., 2012; Zhou et al., 2014). One possible model that we have explored is whether the Golgi outposts that mislocalize to axons in *nudE*^−^ neurons serve as sites of ectopic microtubule nucleation. Although this is an attractive model, other studies have previously found that axonal microtubule polarity is not disrupted by the presence of ectopic Golgi outposts alone (Nguyen et al., 2014; Ye et al., 2007; Zheng et al., 2008). Another possible model is that microtubules of the improper minus-end distal orientation are ectopically translocated into axons in the absence of NudE. We and others have previously suggested that dynein acts as a ‘gatekeeper’ to prevent minus-end distal microtubules from invading axons (Baas and Mozgova, 2012; Zheng et al., 2008), although it remains unknown how dynein carries out this function. Interestingly, recent work has shown that dynein can slide anti-parallel microtubules *in vitro* and that this behavior is likely to be necessary for dynein to organize the mitotic spindle (Tanenbaum et al., 2013), a process which also requires NudE (Raaijmakers et al., 2013). This raises the possibility that dynein might act with NudE to slide minus-end distal microtubules out of the axon. In summary, our experiments with NudE provide insight into the molecular mechanisms by which motor proteins, such as dynein, regulate neuronal morphogenesis.

## MATERIALS AND METHODS

### Transgenic flies

To create the *ppk-EB1–GFP* fly lines, *EB1–GFP* was amplified from genomic DNA isolated from *UAS-EB1–GFP* flies (Rolls et al., 2007) and cloned into an expression vector that contained the *ppk* enhancer (Han et al., 2011). The *ppk-EB1–GFP* expression vector was integrated at *attP-VK37* and *attP-VK20* on the 2nd and 3rd chromosomes, respectively. *UAS-nudE* constructs were generated using the *nudE* cDNA corresponding to isoform A (*Drosophila* Genomics Research Center, Bloomington, IN, USA), which was cloned into the *pACUH* vector. Mutations in *nudE* were introduced through PCR-mediated mutagenesis using PFU Turbo. For the *nudE^ΔC^* mutant constructs (*UAS-nudE^ΔC^* and *genomic-nudE^ΔC^*), a premature stop codon was introduced after amino acid residue 196, changing the sequence from AATAC to TAATAG. We also mutated two amino acid residues (E119A and R130A) that are reported to mediate an interaction with Lis1 (Wang and Zheng, 2011; Zyłkiewicz et al., 2011). The wild-type and mutant constructs were integrated at *attP-VK37* on the 2nd chromosome. The genomic rescue construct was generated by amplifying the *nudE* genomic region from *BAC RP98-7A5* (CHORI, Oakland, CA, USA) with the following primer pair: 5′-CACTCACACAGTTGACATTTGG-3′ and 5′-TGGCATCTGGAAAGGAGTTAGT-3′. This genomic sequence was cloned into *pGE-attB-GMR* using convenient *Nhe*I sites flanking *nudE* and then integrated on the 3rd chromosome at *attP-VK20.* The cloned sequence spans the entire *nudE* transcript, as well as ∼650 bp upstream of the 5′ UTR, including ∼20 bp of the 5′ UTR of the neighboring gene *CG6707*, and ∼420 bp downstream of the 3′ UTR. We generated *UAS-Cherry–NudE* by inserting *mCherry* at the 5′ end of *nudE* in the *pACUH* vector and integrating the construct at *attP-VK37*. We confirmed that the full-length Cherry–NudE fusion protein was expressed by western blotting using an antibody against red fluorescent protein (Clontech, Mountain View, CA, USA) to detect Cherry–NudE in the lysate from the heads of adult *elav-Gal4 UAS-Cherry–NudE* flies. A band migrating at the expected size of the fusion protein indicates that the full-length Cherry–NudE is expressed in neurons.

### Fly stocks and crosses

The following alleles and transgenic lines were used: the protein null allele *nudE^39A^* (Wainman et al., 2009) and *Df(3L)BSC673* (Bloomington Stock Center, Bloomington, IN, USA) were used to eliminate NudE; *γTub23C^A15-2^* and *γTub23C^A14-9^* (Bloomington Stock Center, Bloomington, IN, USA) were utilized to reduce γ-tubulin activity; *UAS-nudE^wild type^, UAS-nudE^ΔC^*, *nudE^genomic-wild-type^*, *nudE^genomic-ΔC^* and *nudE^genomic-E113A,R124A^* were generated in this study (see above for details on their construction); *ppk-CD4–GFP* (Han et al., 2011) and *ppk-EB1–GFP* (this study) were used to visualize neuronal morphology and microtubule polarity, respectively; *UAS-Lis1* (Bloomington Stock Center, Bloomington, IN, USA); *ppk-Gal4* was used to express *UAS-Nod–GFP* (Bloomington Stock Center, Bloomington, IN, USA) and *UAS-Cherry–nudE* (this study), either alone or in combination with *UAS-ManII–GFP* (Ye et al., 2007); to characterize Golgi outpost localization in control and *nudE*^−^ neurons, *UAS-ManII–GFP* was expressed in combination with *UAS-mCD8–GFP* using *Gal4^477^.*

### Image capture and analysis

Live 3rd instar larvae were anesthetized using FlyNap (Carolina Biologicals, Burlington, NC, USA), transferred to grape agar plates to recover and then imaged in a drop of 50% PBS, 50% glycerol solution. Larvae were further immobilized by pressing a cover slip on top of two lines of vacuum grease spacers flanking the animal. The *ppk-Gal4 UAS-Cherry–NudE* flies were not incubated with FlyNap. The larvae were imaged on a Leica SP5 microscope using a HyD GaAsP detector. The imaging and analysis parameters for specific experiments are described below.

#### EB1–GFP and Cherry–NudE

One or two ddaC neurons per animal were imaged for 4–6 minutes. Movies were captured at 1.51 frames/s at a resolution of 1024×512 pixels using a 40×1.3 NA oil immersion objective. EB1–GFP comets and Cherry–NudE particles in axons and dendrites within 100 µm of the cell body were analyzed; in dendrites, only segments between branch points were analyzed. Movies were stabilized using the FIJI Image Stabilizer plugin. For EB1–GFP analysis, kymographs for each axon or dendrite were generated using ImageJ or FIJI. Comet trajectories were manually traced in ImageJ and the coordinates were exported to Excel to obtain velocity information. For Cherry–NudE, analysis was completed using the Imaris particle tracking wizard. The following parameters were used – only particles >1 µm in diameter were analyzed. Particles that moved more than 2 µm every 0.66 s (1 frame=0.66 s) and were in focus for at least six frames were considered tracks. Particles were classified as stationary if they did not move faster than 0.2 μm/s or more than 1.5 μm.

#### Dendrite arborization

Images of ddaC sensory neurons were captured at a resolution of 2048×2048 pixels using a 20×0.7 NA oil immersion objective. *z*-stacks that were 10-40-μm thick were analyzed using the Imaris software program FilamentTracer. Signals from neighboring neurons were masked so that those from only a single neuron were quantified. The FilamentTracer was used to calculate dendrite length and the number of branch points, and to perform Sholl analysis to quantify the distribution of branches within a dendrite arbor. Data was exported to Excel and statistically analyzed.

### Statistical analysis

Data were analyzed using Student's unpaired *t*-tests to compare individual samples and one-way ANOVA with Tukey (a=0.01 was used to determine whether a significant difference existed) and *t*-tests post hoc to compare multiple samples. Percentages were analyzed using a Fisher's exact test. Significance levels are represented as follows: n.s. (not significant), *P=0.05–0.01, **P=0.01–0.001, ***P=0.001–0.0001 and ****P<0.0001. Error bars indicate s.d.
